# Quantitative Analysis of Organic Liquid Three-Component Systems: Near-Infrared Transmission versus Raman Spectroscopy, Partial Least Squares versus Classical Least Squares Regression Evaluation and Volume versus Weight Percent Concentration Units

**DOI:** 10.3390/molecules24193564

**Published:** 2019-10-01

**Authors:** Hui Yan, Yue Ma, Zhixin Xiong, Heinz W. Siesler, Liang Qi, Guozheng Zhang

**Affiliations:** 1 School of Biotechnology, Jiangsu University of Science and Technology, Zhenjiang 212018, China; mayue15751773676@163.com (Y.M.); justqiliang@sina.com (L.Q.); zgzsri@163.com (G.Z.); 2 College of Light Industry Science and Engineering, Nanjing Forestry University, Longpan Road 159, Nanjing 210037, China; Leo_xzx@njfu.edu.cn; 3 Department of Physical Chemistry, University of Duisburg-Essen, Schützenbahn 70, D 45117 Essen, Germany; hw.siesler@uni-due.de

**Keywords:** near-infrared (NIR) spectroscopy, Raman spectroscopy, organic liquid three-component mixtures, molecular interactions, partial least squares (PLS) regression, classical least squares (CLS) regression, volume/weight percent concentration units

## Abstract

The band shapes and band positions of near-infrared (NIR) and Raman spectra change depending on the concentrations of specific chemical functionalities in a multicomponent system. To elucidate these effects in more detail and clarify their impact on the analytical measurement techniques and evaluation procedures, NIR transmission spectra and Raman spectra of two organic liquid three-component systems with variable compositions were analyzed by two different multivariate calibration procedures, partial least squares (PLS) and classical least-squares (CLS) regression. Furthermore, the effect of applying different concentration units (volume percent (%V) and weight percent (%W) on the performance of the two calibration procedures have been tested. While the mixtures of benzene/cyclohexane/ethylbenzene (system 1) can be regarded as a blended system with comparatively low molecular interactions, hydrogen bonding plays a dominant role in the blends of ethyl acetate/1-heptanol/1,4-dioxane (system 2). Whereas system 1 yielded equally good calibrations by PLS and CLS regression, for system 2 acceptable results were only obtained by PLS regression. Additionally, for both sample systems, Raman spectra generally led to lower calibration performance than NIR spectra. Finally, volume and weight percent concentration units yielded comparable results for both chemometric evaluation procedures.

## 1. Introduction

Due to the different physical excitation mechanisms of mid-infrared (MIR)/NIR and Raman spectra, molecular interaction in multicomponent systems (e.g., hydrogen bonding) can affect these types of vibrational spectra to different extents. Hydrogen bonding plays a vital role in the molecular interaction of OH and NH groups with carbonyl or ether functionalities and has significant footprints in the MIR spectra of multicomponent systems with relevant chemistry [1,2,3,4,5]. These spectral effects, however, are not only observed with fundamental vibrations in the mid-infrared region but also occur in the overtone and combination band region of the near-infrared [6]. The application of NIR transmission spectroscopy as a counterpart of Raman spectroscopy has the advantage, to circumvent on the one hand the sample thickness limitations of MIR transmission spectroscopy for liquid mixtures and to avoid on the other hand the distortion and intensity changes of MIR absorption bands by the attenuated total reflection (ATR) technique [7].

Calibrations by CLS regression have often proved to be of excellent performance for multicomponent systems with no or small molecular interactions (e.g., gas analytical applications) [8,9]. However, no reports are available on the CLS calibration performance for multicomponent systems with strong molecular interactions. Thus, in this work, apart from comparing the impact of molecular interactions in liquid multicomponent systems on the results obtained by the two different types of spectroscopies, a further topic of this publication are the effects of these structural phenomena on the performance of two different multivariate evaluation routines (PLS and CLS regression) for the quantitative analysis of the investigated liquid three-component systems. Last but not least—and with reference to previous studies by Mark et al. [10,11,12,13]—we have tried to shed light on the consequences of using different concentration units (%V and %W) for the quantitative analysis of the described multicomponent systems.

## 2. Results and Discussion

### 2.1. Spectral Characteristics

#### 2.1.1. NIR Spectra

**Figure 1:**
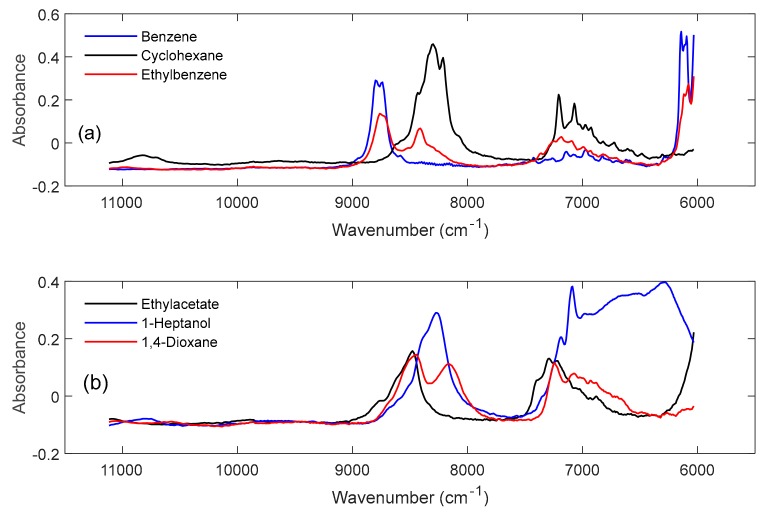
NIR spectra of the pure components of system 1 (**a**) and system 2 (**b**).

The NIR spectra of the pure components of system 1 and system 2 are shown in Figure 1a,b, respectively. The NIR spectra of system 1 show a clear separation into aromatic and aliphatic/cycloaliphatic overtone and combination bands [14]. Thus, the bands between 6000–6200 cm^−1^ and in the wavenumber range 8500–9000 cm^−1^ can be assigned to the 1st and 2nd overtones, respectively, of the ν(CH)_ar_ vibrations of benzene and ethylbenzene, whereas the 8000–8500 cm^−1^ region is characteristic of the 2nd overtones of the ν(CH_2_) and ν(CH_3_) vibrations of cyclohexane and ethylbenzene.

The more intense bands in the 7000 cm^−1^ range (black and red spectra) belong to 2xν(CH_2_) + δ(CH_2_) and 2xν(CH_3_) + δ(CH_3_) combination vibrations of cyclohexane and ethylbenzene, whereas the very weak band complex (blue spectrum), can be assigned to combination bands of the 2xν(CH)_ar_ + δ(CH)_ar_ out-of-plane vibrations of benzene.

The band assignment of the aliphatic functionalities in the NIR spectra of the pure components of system 2 is analogous to system 1 for 1,4-dioxane and ethyl acetate whereas it becomes more complex for 1-heptanol due to the overlap with OH-specific absorption bands. Particularly noticeable are the intense, broad absorption bands of the 2xν(OH) + δ(OH) (8000–8500 cm^−1^) combination and 2xν(OH) (6000–7000 cm^−1^) 1st overtone vibrations of the hydrogen-bonded OH-functionalities.

**Figure 2:**
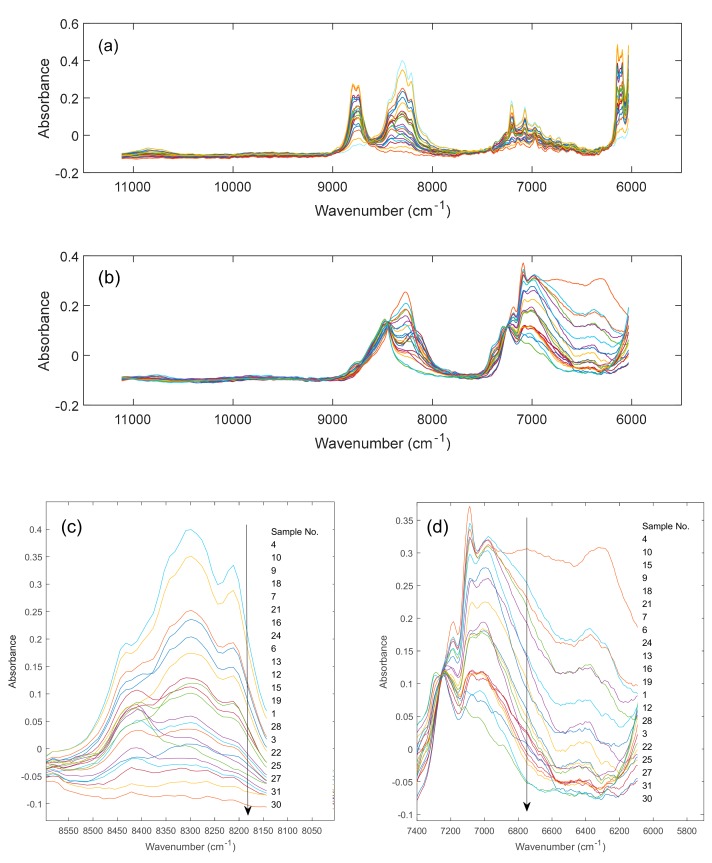
NIR spectra (11,000–6000 cm^−1^) of the different three-component solvent mixtures of system 1 (**a**) and system 2 (**b**). In (**c**,**d**) enlargements for specific wavenumber ranges of (**a**,**b**) (see text) are shown.

The NIR spectra of all solvent blends of system 1 and system 2 with variable compositions are shown for the total available wavenumber range in Figure 2a,b, respectively. For better visualization, enlargements of the wavenumber range 8600–8150 cm^−1^ (system 1) and 7400–6100 cm^−1^ (system 2) are included in (c) and (d), respectively. Here, the dipole moment relevant differences in molecular interaction between the individual components of system 1 and system 2 lead to an interesting phenomenon. While the absorption bands of system 1 are more or less wavenumber invariant and only vary in intensity as a function of changing compositions, the absorption bands of system 2 not only reflect intensity fluctuations but also undergo drastic band shifts as a function of varying compositions. This can best be seen in Figure 2d, where the change in hydrogen bonding strength as a function of blend composition (the hydrogen bonding strength is different for the two hydrogen bonding acceptors) leads to drastic band shifts in the wavenumber range 6100–6600 cm^−1^.

#### 2.1.2. Raman Spectra

**Figure 3:**
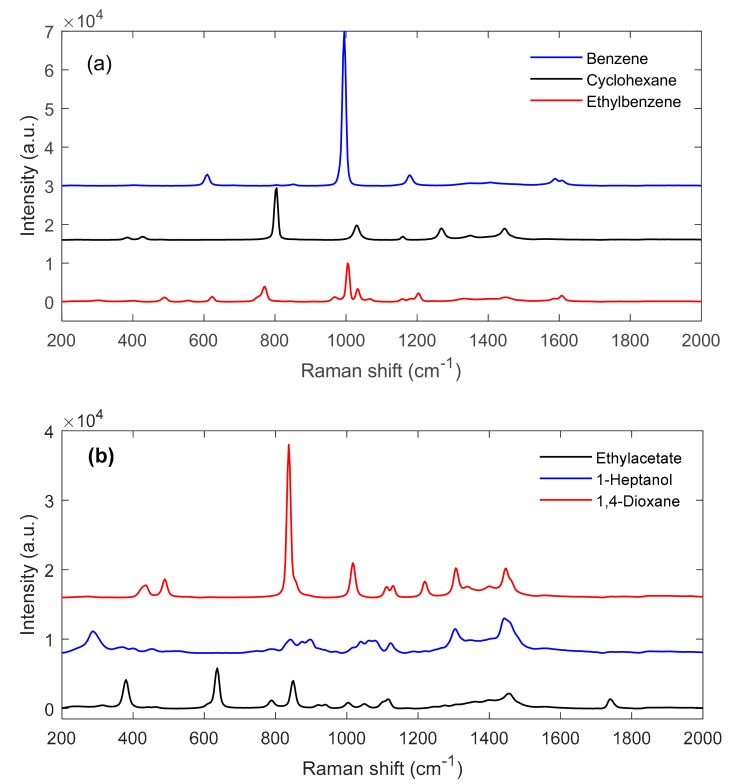
Raman spectra of the pure components of system 1 (**a**) and system 2 (**b**).

The Raman spectra of the pure components of system 1 and system 2 are shown in Figure 3a,b, respectively. With the exception of 1-heptanol, in contrast to the NIR spectra, the Raman spectra are characterized by comparatively narrow and well-separated signals.

**Figure 4:**
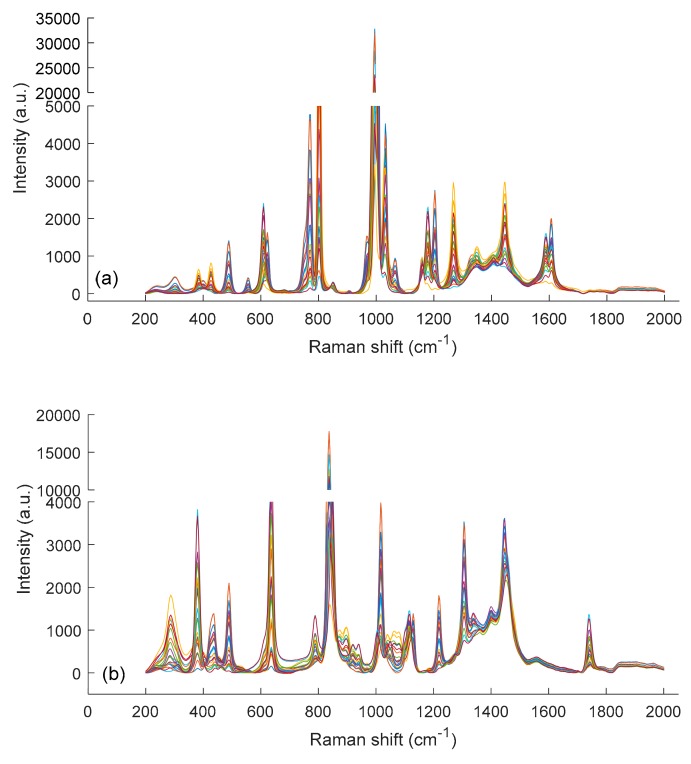

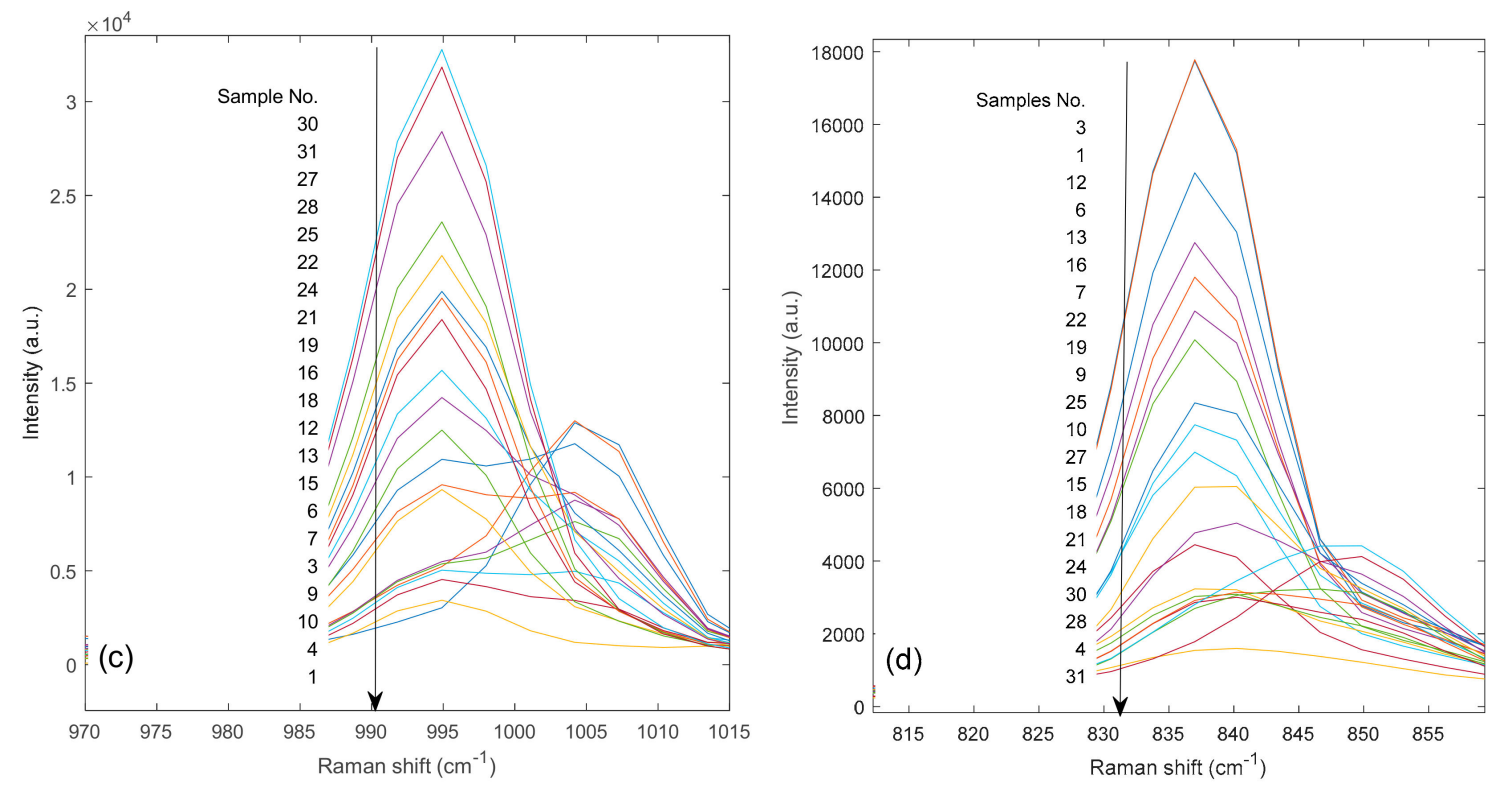
Raman spectra (200–2000 cm^−1^) of the different three-component solvent mixtures of system 1 (**a**) and system 2 (**b**). In (**c**,**d**) enlargements for specific wavenumber ranges of (**a**,**b**) (see text) are shown.

In system 1, the most intense signals originate from ring-breathing vibrations around 1000 cm^−1^ (benzene and ethylbenzene) and 800 cm^−1^ (cyclohexane), respectively. The last-mentioned signal is also observed in the spectrum of 1,4-dioxane (system 2). More detailed band assignments are available in a recently published book by G. G. Hoffmann [15]. The Raman spectra of all variable-composition solvent blends of system 1 and system 2 are shown in Figure 4a,b respectively. Similar to the NIR spectra of system 1 and unlike the NIR spectra of system 2, the Raman spectra of both mixture systems reflect primarily composition-dependent intensity changes with only minor band shifts. This phenomenon is accentuated in the enlargement of Figure 4c for the wavenumber range 985–1015 cm^−1^ (system 1) and the enlargement of Figure 4d for the wavenumber range 830–860 cm^−1^ (system 2).

Generally, the qualitative comparison of NIR and Raman spectra has to take into account their different excitation conditions. Thus, the dipole moment change of mechanically anharmonic oscillators with significant mass differences—such as the OH functionality of 1-heptanol—is susceptible to hydrogen bonding and leads to substantial spectral changes in the NIR spectra of the variable-composition blends. The corresponding Raman spectra, on the other hand, originate from changes in the polarizability, i.e., the measure for the simplicity to deform the electron envelope of a molecule during vibration. In a separate publication it will be shown, that although the impact of the composition-dependent polarizability changes of the skeletal, ring breathing, and ring deformation vibrations of system 1 is in a first inspection less obvious for the Raman spectra, closer examination also reveals intermolecular interactions for the cycloaliphatic and aromatic components of this system.

### 2.2. Comparison of the Calibration Performance

#### 2.2.1. PLS/CLS Calibrations of NIR Spectra

As shown by the root mean square error (RMSE) and R square (R^2^) values for system 1 in Table 1, the NIR-based CLS calibrations are of similar high quality as the PLS calibrations. With only two factors, the PLS calibrations also require the lowest possible number of factors for a 3-component system. For system 2, with strongly interacting components, however, CLS shows generally lower calibration performance than the corresponding PLS calibration. For the hydrogen bonding acceptors (ethyl acetate and 1,4-dioxane), the interactions with the hydrogen bonding donor (1-heptanol) are compensated by extra factors in the PLS calibrations. At this point, however, the lower number of factors required for the hydrogen bonding donor (only 2), cannot be explained.

Thus, the CLS calibration method is preferably best applied to weakly or non-interacting mixture systems, where the NIR spectra of any composition can be almost perfectly reconstructed from pure-component spectra. In the CLS calibration, the spectrum is modeled as a weighted sum of the pure component spectra and the baseline function. For many simple mixtures, this method may be accurate enough, but it is not able to model molecular interaction effects such as peak broadening and substantial peak shifts [16,17,18]. Thus, the CLS model performance is high for system 1 and low for system 2.

#### 2.2.2. PLS/CLS Calibrations of Raman Spectra

In Table 2, the calibration parameters obtained for the Raman spectra have been summarized. Most strikingly almost all PLS and CLS calibration parameters for both sample systems are of lower quality. The reason may be, that the S/N ratio of the NIR instrument used in the present investigations is much higher (10,000:1) than that of the Raman instrument (1000:1). Furthermore, unlike the results for the NIR spectra, the PLS calibrations of the Raman spectra clearly outperform the CLS calibrations for both sample systems. This is a consequence of the fact, that contrary to NIR spectroscopy, the Raman technique does slightly reflect spectral changes by molecular interactions of the mixture components of system 1 in the form of small band shifts of skeletal and ring breathing vibrations. Using a similar approach of pure-spectra reconstruction with the Raman spectra of system 1 as described in detail in a previous publication for NIR spectra [5], these interaction effects are observed as dispersion shaped signals.

#### 2.2.3. Comparison of Calibration Performance Obtained with Volume and Weight Percent Concentrations

As described in Section 2.2, the samples were prepared by mixing the individual components by volume percentage. Subsequently, the density of each sample was determined, and the volume percentage concentrations (%V) were transformed into weight percentage concentrations (%W), and the volumes of the mixture solutions were calculated. From this procedure it was found that the differences in the actual volumes of the mixture samples and the sum of the volumes of the pure components used for sample preparation was l minimal, and the coefficients of variation for the actual volumes of the mixture samples were 0.43% and 0.51% for system 1 and system 2, respectively. Furthermore, in their publications [10,11,12,13] Mark et al. claimed, that for NIR calibrations the use of volume percentage—corresponding to the scaled volume fraction concentration unit of Beer’s law—is the better approach than weight percentage. In what follows we will show, that this statement is not supported by the results of the investigated multicomponent systems.

In Table 1 and Table 2 not only the results for both spectroscopic techniques and calibration procedures have been summarized, but also the parameters derived for both concentration units have been included. Although the rows with the concentration units that yielded the better calibration results have been highlighted in bold typeface in the Table 1 and Table 2, it has to be clearly stated, that the differences in the RMSE and R^2^ values, derived with the different concentration units, are rather small. Furthermore, the assignment of compounds with superior calibration does not allow for the definition of general rules based on specific chemical or physical phenomena, which could be eventually used to improve calibration performance. Therefore, volume and weight percent concentration units should be treated to perform equally for the respective calibration procedures with both spectroscopic techniques and sample systems under investigation.

## 3. Materials and Methods

### 3.1. Chemicals

Six organic liquids were selected to prepare 21 calibration samples and 10 test samples (30 mL each) for each of the two mixture systems with variable concentrations: benzene, cyclohexane, and ethylbenzene (system 1), and ethyl acetate, 1-heptanol, and 1,4-dioxane (system 2). The six chemicals were purchased from Sinopharm Chemical Reagent Co. Ltd. (Shanghai, China).

### 3.2. Determination of Volume and Weight Percentage Concentrations

The densities of the pure components and the individual solvent mixtures of the two sample systems were determined with a calibrated 25 mL volumetric flask. Based on these values, the weight percentages (%W) of the individual components in the different solvent mixtures were calculated. In Table 3, the volume and weight percentage compositions of system 1 and system 2 are summarized.

### 3.3. Instrumentation

The NIR spectra of the solvent mixtures were measured in the wavenumber range 11,117–5853 cm^−1^ with a NIRQuest512 spectrometer (Ocean Optics, Inc., Orlando, FL, USA) based on a grating monochromator, an uncooled InGaAs array detector, equipped with a HL-2000 light source (Ocean Optics, Inc., Orlando, FL, USA), and coupled to an optical fiber. The instrument has a signal-to-noise (S/N) ratio of 10,000:1. The liquid samples were measured in a 2 mm path length transmission cell with an integration time of 14 ms by accumulating 20 scans with a mean spectral resolution of 20.6 cm^−1^. Each sample was measured in triplicate, and the mean spectrum was calculated as the final result.

The Raman spectra of the solvent mixtures were measured with a QE65 Pro Raman spectrometer (Ocean Optics, Inc., Orlando, FL, USA), that was equipped with a grating monochromator, a cooled InGaAs array detector and with an optical fiber coupled to a 180 mW Turnkey Raman Laser with 785 nm excitation (Innovative Photonic Solutions, Monmouth Junction, NJ, USA). The instrument has an S/N ratio of only 1000:1. The samples were placed in a GC glass bottle (φ: 10 mm, height: 32 mm, Zhejiang Aijiren Technology Co., Ltd., Zhejiang, China) and measured in the 136–2200 cm^−1^ wavenumber range with an integration time of 6 s by accumulating five scans with a mean spectral resolution of 5.8 cm^−1^. Each sample was measured in triplicate, and the mean spectrum was used for further evaluations.

### 3.4. Chemometric Data Analysis

The PLS toolbox 6.21 (Eigenvector Research, Inc., Manson, WA, USA) was used for chemometric data analysis. For this purpose, the original NIR and Raman spectra were truncated to the wavenumber range 11,117–6033 cm^−1^ and 200–2000 cm^−1^, respectively. As an additional data pretreatment, the baseline correction was applied, and then the calibration spectra were subjected to PLS and CLS calibrations with a leave-one-out (LOO) internal cross-validation to select the optimum number of factors.

### 3.5. Calibration Statistics Analysis

Calibration statistics included the R square of calibration (*R^2^_C_*), the R square of cross-validation (*R^2^_CV_*), the R square of prediction (*R^2^_P_*), the root mean square error of calibration (*RMSEC*), the root mean square error of cross-validation (*RMSECV*), and the root mean square error of prediction (*RMSEP*). The R square is used to describe the linear correlation between the predicted values and the measured values. The higher the *R^2^p* and the closer it is to *R^2^c*, the higher is the correlation between the predicted and the actual values, and the robustness of the model. The *RMSEC*, *RMSECV*, and *RMSEP* were used to evaluate the feasibility of the calibration model [19]. The lower the *RMSEP* and the closer it is to the *RMSEC*, the stronger is the predictive ability and the robustness of the calibration model [20].

## 4. Conclusions

The mixtures of benzene/cyclohexane/ethylbenzene (system 1) can be regarded as a blended system with comparatively low molecular interactions, whereas hydrogen bonding plays a dominant role in the blends of ethyl acetate/1-heptanol/1,4-dioxane (system 2).

The calibration results evaluated by PLS and CLS regression with the NIR and Raman spectra of the investigated 3-component systems 1 and 2 taking into consideration volume percent and weight percent concentration units allow to draw the following conclusions:

(1) Multicomponent systems—as system 1 in the present work—that do not induce significant band shifts in the NIR spectra of different blend compositions yield equally good calibrations by PLS and CLS regression.
(2) Multicomponent systems with large spectral signatures due to molecular interactions by hydrogen bonding—like system 2 in the present work—should only be calibrated by PLS regression, because the negative effect of the molecular interactions can be efficiently compensated by the increase of the number of factors.
(3) For both sample systems, Raman spectra led to lower calibration performance than NIR spectra. Specifically, for system 1—the aromatic and cycloaliphatic 3-component system—a significant deterioration of calibration results by PLS and CLS regression was observed.
(4) The hypothesis, that volume percent should be preferentially used as the concentration unit for the calibration of liquid multicomponent systems could not be confirmed by the presented results. For both spectroscopic measurement techniques as well as chemometric calibration procedures volume and weight percent concentration units yielded comparable results.

## Figures and Tables

**Table 1:** The calibration and test results obtained with PLS and CLS regressions of the NIR spectra of system 1 and system 2 based on %V and %W concentrations (the bold values highlight the higher-quality results with reference to the concentration dimensions).

Concentration Parameters	Factors	PLS						CLS					
		*RMSEC*	*RMSECV*	*RMSEP*	*R^2^_C_*	*R^2^_CV_*	*R^2^_P_*	*RMSEC*	*RMSECV*	*RMSEP*	*R^2^_C_*	*R^2^_CV_*	*R^2^_P_*
Benzene (%V)	2	0.8	1.0	0.9	0.970	0.999	0.999	0.8	1.1	0.7	0.999	0.998	0.999
**Benzene (%W)**	**2**	**0.5**	**0.6**	**0.5**	**1.000**	**1.000**	**1.000**	**0.5**	**0.6**	**0.4**	**1.000**	**0.999**	**1.000**
Cyclohexane (%V)	2	0.6	0.7	0.6	1.000	0.999	0.999	0.6	0.7	0.6	0.999	0.999	0.999
**Cyclohexane (%W)**	**2**	**0.4**	**0.6**	**0.5**	**1.000**	**1.000**	**1.000**	**0.5**	**0.6**	**0.5**	**1.000**	**0.999**	**1.000**
**Ethylbenzene (%V)**	**2**	**0.6**	**0.7**	**0.6**	**0.999**	**0.999**	**0.999**	**0.8**	**0.9**	**0.4**	**0.999**	**0.999**	**1.000**
Ethylbenzene (%W)	2	0.8	1.0	0.9	0.999	0.999	0.998	0.8	1.0	0.5	0.999	0.999	1.000
**Ethyl acetate (%V)**	**4**	**0.4**	**0.7**	**0.6**	**1.000**	**0.999**	**0.999**	**2.2**	**2.7**	**1.3**	**0.993**	**0.990**	**0.998**
Ethyl acetate (%W)	4	0.5	0.8	0.6	1.000	0.999	0.999	2.6	3.7	1.2	0.991	0.982	0.999
1-Heptanol (%V)	2	0.9	1.2	0.8	0.998	0.998	0.996	1.1	1.3	1.0	0.997	0.995	0.997
**1-Heptanol (%W)**	**2**	**0.5**	**0.6**	**0.5**	**1.000**	**0.999**	**0.998**	**0.7**	**1.0**	**0.7**	**0.998**	**0.997**	**0.997**
1,4-Dioxane (%V)	*4*	0.7	1.1	1.0	0.999	0.999	0.998	3.9	4.8	3.9	0.980	0.971	0.989
**1,4-Dioxane (%W)**	*4*	0.7	1.1	1.0	0.999	0.999	0.998	**3.8**	**4.9**	**3.7**	**0.982**	**0.969**	**0.991**

**Table 2:** The calibration and test results obtained with PLS and CLS regressions of the Raman spectra of system 1 and system 2 based on %V and %W concentrations (the bold values highlight the higher-quality results with reference to the concentration dimensions).

Concentration Parameters	Factors	PLS						CLS					
		*RMSEC*	*RMSECV*	*RMSEP*	*R^2^_C_*	*R^2^_CV_*	*R^2^_P_*	*RMSEC*	*RMSECV*	*RMSEP*	*R^2^_C_*	*R^2^_CV_*	*R^2^_P_*
Benzene (% V)	3	1.8	2.4	2.3	0.995	0.993	0.991	3.1	3.5	5.7	0.987	0.983	0.969
**Benzene (% W)**	**3**	**1.7**	**2.2**	**1.8**	**0.996**	**0.994**	**0.995**	**3.0**	**3.4**	**5.3**	**0.988**	**0.985**	**0.973**
Cyclohexane (%V)	3	1.3	1.6	1.3	0.998	0.996	0.997	2.0	2.3	1.4	0.994	0.992	0.998
**Cyclohexane (% W)**	**3**	**1.1**	**1.3**	**0.8**	**0.998**	**0.997**	**0.999**	**1.7**	**1.9**	**1.2**	**0.996**	**0.994**	**0.998**
**Ethylbenzene (% V)**	3	1.6	2.1	1.2	0.996	0.995	0.997	2.4	2.7	1.8	0.993	0.990	0.995
Ethylbenzene (% W)	3	1.7	2.2	1.4	0.996	0.994	0.996	2.6	3.0	2.1	0.991	0.988	0.993
Ethyl acetate (% V)	2	1.9	2.3	2.0	0.995	0.994	0.994	2.4	3.4	2.6	0.991	0.983	0.996
**Ethyl acetate (% W)**	**2**	**1.7**	**2.1**	**1.8**	**0.996**	**0.994**	**0.992**	**2.3**	**3.2**	**2.5**	**0.991**	**0.984**	**0.996**
**1-Heptanol (% V)**	**3**	**1.1**	**1.4**	**1.7**	**0.998**	**0.997**	**0.995**	**1.9**	**2.0**	**1.9**	**0.995**	**0.994**	**0.993**
1-Heptanol (% W)	3	1.4	1.8	2.2	0.997	0.995	0.992	2.1	2.4	2.4	0.993	0.990	0.988
**1,4-Dioxane (% V)**	**3**	**1.0**	**1.3**	**1.1**	**0.999**	**0.998**	**0.997**	**1.7**	**2.1**	**1.9**	**0.996**	**0.994**	**0.999**
1,4-Dioxane (% W)	3	1.3	1.7	1.2	0.998	0.997	0.997	2.3	2.6	2.1	0.993	0.992	0.995

**Table 3:** Volume and weight percentage (%V and %W) compositions of the calibration and test samples (total volume 30 mL) of the two mixture systems.

Sample Set	Sample No.	Benzene	Cyclohexane	Ethylbenzene	Benzene	Cyclohexane	Ethylbenzene	Ethyl Acetate	1-Heptanol	1,4-Dioxane
		Ethyl Acetate	1-Heptanol	1,4-Dioxane						
		(% V)			(% W)			(% W)		
**Calibration set**	1	2.50	7.50	90.00	2.54	6.77	90.70	2.22	6.19	91.59
	3	7.50	2.50	90.00	7.57	2.24	90.19	6.64	2.06	91.31
	4	7.50	90.00	2.50	8.33	88.91	2.76	7.97	88.98	3.05
	6	10.00	30.00	60.00	10.39	27.71	61.90	9.37	26.15	64.48
	7	10.00	40.00	50.00	10.50	37.35	52.15	9.57	35.59	54.85
	9	10.00	60.00	30.00	10.73	57.28	31.99	9.98	55.69	34.33
	10	10.00	70.00	20.00	10.86	67.58	21.57	10.20	66.41	23.39
	12	20.00	10.00	70.00	20.32	9.04	70.65	18.25	8.49	73.26
	13	20.00	20.00	60.00	20.54	18.26	61.20	18.62	17.32	64.06
	15	20.00	70.00	10.00	21.70	67.53	10.78	20.71	67.41	11.87
	16	30.00	20.00	50.00	30.78	18.25	50.97	28.32	17.56	54.12
	18	30.00	60.00	10.00	32.16	57.20	10.65	30.84	57.37	11.79
	19	40.00	20.00	40.00	41.02	18.24	40.75	38.29	17.80	43.91
	21	40.00	50.00	10.00	42.37	47.10	10.52	40.83	47.46	11.71
	22	50.00	10.00	40.00	50.69	9.02	40.29	47.54	8.84	43.61
	24	50.00	40.00	10.00	52.35	37.25	10.40	50.68	37.70	11.62
	25	60.00	10.00	30.00	60.79	9.01	30.20	57.86	8.97	33.17
	27	70.00	10.00	20.00	70.88	9.00	20.12	68.47	9.10	22.43
	28	70.00	20.00	10.00	71.63	18.20	10.17	69.95	18.59	11.46
	30	90.00	2.50	7.50	90.29	2.23	7.47	89.18	2.30	8.52
	31	90.00	7.50	2.50	90.77	6.73	2.50	90.14	6.99	2.87
**Test set**	2	2.50	90.00	7.50	2.78	88.94	8.28	2.64	88.29	9.07
	5	10.00	10.00	80.00	10.17	9.04	80.79	9.01	8.38	82.62
	8	10.00	50.00	40.00	10.62	47.20	42.18	9.77	45.42	44.81
	11	10.00	80.00	10.00	10.98	78.11	10.91	10.43	77.61	11.96
	14	20.00	30.00	50.00	20.76	27.69	51.55	19.01	26.51	54.48
	17	30.00	40.00	30.00	31.45	37.30	31.25	29.53	36.61	33.86
	20	40.00	30.00	30.00	41.46	27.65	30.89	39.10	27.27	33.63
	23	50.00	30.00	20.00	51.79	27.63	20.58	49.59	27.67	22.74
	26	60.00	30.00	10.00	62.10	27.61	10.28	60.38	28.08	11.54
	29	80.00	10.00	10.00	80.95	9.00	10.05	79.39	9.23	11.38
